# The Role of Cardiovascular Risk Assessment in Preventive Medicine: A Perspective from Portugal Primary Health-Care Cardiovascular Risk Assessment

**DOI:** 10.1155/2020/1639634

**Published:** 2020-01-30

**Authors:** Paulo Santos

**Affiliations:** ^1^Department of Medicine of Community, Information and Health Decision Sciences (MEDCIDS), Faculty of Medicine, University of Porto, Porto, Portugal; ^2^CINTESIS—Center for Health Technology and Services Research, University of Porto, Porto, Portugal

## Abstract

The cardiovascular diseases are the leading cause of death in the world, especially because of myocardial infarction and stroke. Their beginning, however, starts many years earlier with the atherosclerotic process due to the cardiovascular risk factors, with different weights in the global risk. Our aim is to review the utilization of risk estimators in primary health care, through a comprehensive review of the literature and official national and international health data (OECD and WHO). The risk estimators aim to integrate the partial information of each factor in a global calculation able to help towards a better clinical reasoning in primary prevention. Besides the variables in the mathematical algorithm, estimators must consider also the factors not in the equation, but significant for decision making. Risk estimators are crucial in prevention, allowing to classify the risk in practical categories easy to use and to benefit the decision-making, more than trying to guess what will happen to the patient.

## 1. Introduction

**Figure 1:**
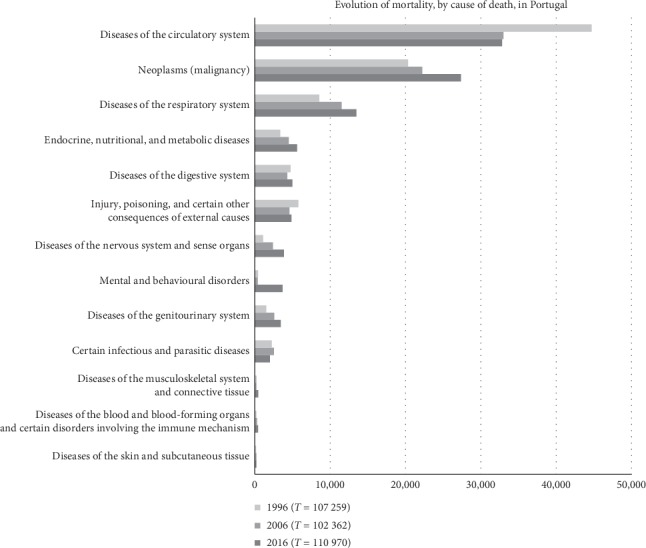
Absolute mortality in Portugal between 1996–2016 (based on official data from National Statistics Institute-INE, 2018).

Cardiovascular diseases are currently the leading cause of death in the world. In Portugal, in 2016, they represented more than 32,000 deaths, about one-third of the total, with the highest prevalence of cerebrovascular disease (Figure 1).

But the scenery was not always like this. At the beginning of the 20^th^ century, infectious diseases were preponderant [1]. This situation has changed with the socioeconomic transformation in developed countries, especially after the 2^nd^ Great War, by the improvement in the hygienic conditions, in the distribution of potable water and in the collection of the wastes and sewages, by the democratization of the access to education and employment, and by the significant improvement in the economic conditions of the population. The epidemiological transition verified during the last century led to the fall in infectious diseases, with the reduction in early mortality and the improvement of the life expectancy. Consequently, we saw a raise in the noncommunicable diseases, mostly related to behavioral options and lifestyles, reflected in the chronic and degenerative diseases [2].

The deterministic model of Henle–Koch, described in the nineteenth century, tried to explain the causality of diseases from the infectious point of view. However, it proved to be too simplistic in new epidemiological situation, and now it does not explain the chronic illnesses, where several factors compete for the same effect, or, on the other hand, where different effects arise from the context of an apparent single cause. This new probabilistic thinking is structured by Hill in the multifactorial causality model [3] and forced the review of the postulates by Evans [4], including probabilistic thought and incorporating it into the medical decision.

In the case of cardiovascular diseases, the evolution of events is well known from the normal artery to the point of critical atherosclerosis with rupture of a plaque [5]. If we think on its natural history, we may find several cutting points where we can intervene to modelling the sequence of events and even prevent their appearance. On the edge, if we identify all the factors that explain the variation, we can anticipate the effect and avoid the disease. In the present state of the art, the model is not completely established. Risk factors are known (Table 1) [6], but the way they interact with each other remains still unclear, which makes it difficult to manage the available information.

The absolute prevalence of each risk factor is also important, as it conditions the probability of an event and the way it manifests. The higher the prevalence of arterial hypertension, tobacco, diabetes, or lipid disorders in the population is, the greater will be the final impact on cardiovascular disease [7]. Moreover, the way the different risk factors combine with each other does not follow a linear random distribution. They present clusters of cardiovascular risk: diabetes is more prevalent in hypertensive patients than in general population, and the same for several other risk factors, depending on patients' metabolic conditions. This conjunction of different risk factors does not result in the sum of the absolute risks but in an exponentiation that conditions a total risk significantly higher than what would be expected by the simple univariate analysis [8,9].

This risk factors gambling justifies that Portugal is a country with very low incidence of coronary disease but presents high incidence of stroke.

The aim of this article is to review the main tools to assess the cardiovascular risk and the way the global risk may be used and interpreted in primary health care.

## 2. What Is the Cardiovascular Risk of Our Patient?

When we address the risk factors for cardiovascular disease, we are actually anticipating the treatment from the disease (event) to a nondisease phase. It is crucial to be sure about the evidence of the effectiveness to support the intervention. Between 40 and 69 years, for instance, a difference of 20 mm Hg in systolic blood pressure is associated with more than two times the risk of death by stroke and about 2 times by ischemic heart disease [10]. A decrease of 5 mm Hg, on the other hand, is associated to a reduction of 14% of stroke deaths, 9% of ischemic heart disease, and 7% in all-cause mortality [11].

**Figure 2:**
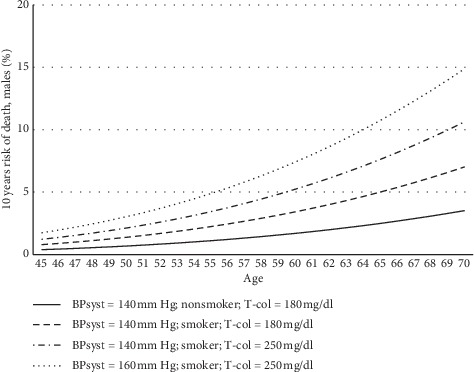
Impact of main cardiovascular risk factors in 10 years' probability of death in males, according to SCORE-risk calculator of European Society of Cardiology [12]. BPsyst: Systolic blood pressure (mm Hg); T-col: Total Cholesterol (mg/dl).

We know the difficulty of adjusting the different risk factors and their relative weighting in a specific patient.  Figure 2 shows the 10 years' risk of death through the age, by the variation of several risk factors in males. Although the general appearance shows an increase, it is hard to gamble the different factors in practice with the patient.

This difficulty made it necessary to define algorithms capable of predicting the probability of occurrence of the event and to help in the decision of the medical intervention, aiming to effectively change the natural history of the atherosclerotic process to prevent cardiovascular disease.

After the shock of the premature death of President Franklin D Roosevelt, in 1945, a study was designed to identify the common factors and characteristics contributing to the cardiovascular disease, following its development in a cohort of healthy individuals over a long period of time [13].

The Framingham study begun in 1948 and included 5,209 men and women from Framingham, a small town in Massachusetts, USA. It introduced much of the current knowledge about the cardiovascular risk factors: tobacco, lipid disorders, high blood pressure, electrocardiographic abnormalities, menopause, atrial fibrillation, overweight, and obesity, among many others associated to the increase of cardiovascular events, and protective factors as physical activity and HDL-cholesterol were also established.

The initial cohort was enhanced in 1971, including the descendants (5,124 sons and daughters) of first participants, and in 2002, included also their 4,095 grandchildren. The research extends now to genetics and epigenetics, describing hundreds of new genes related to main cardiovascular diseases and their precursors or risk factors.

In 1994, the first non-Caucasian cohort was added, including 507 participants OMNI (African Americans, Hispanics, Asians, Indians, Native Americans, and Pacific Islanders) and 410 more, later in 2003 [14].

The risk calculators are one of the outcomes from the Framingham study. The most known is the Risk Score for Prediction of Cardiovascular Diseases [15]. It estimates the 10 years risk of cardiovascular disease or death (coronary death, acute myocardial infarction, coronary ischemia, angina, ischemic or hemorrhagic stroke, transient ischemic attack, peripheral arterial disease, and congestive heart failure) in individuals from 30 to 74 years of age in primary prevention, using age, sex, smoking habits, systolic blood pressure, diabetes mellitus, and total cholesterol and HDL in the total model or body mass index, in the simplified one.

There are many other algorithms for risk estimation:

- The Globorisk is an extension of Framingham calculator and 7 more prospective studies. It estimates the 10 years risk of fatal cardiovascular disease between 20- and 80-year-old people [16].
- The American College of Cardiology/American Heart Association Task Force proposed a new *Pooled Cohort ASCVD Risk Equations* [17] allowing the estimation of cardiovascular disease between 40- and 79-year- old people adjusted for sex and race (Caucasians and African Americans). The variables in the model are age, total cholesterol, HDL-cholesterol, systolic blood pressure (including treated and nontreated patients), diabetes mellitus, and smoking habits.
- The Reynolds estimator adapts the calculation formula to women by entering age, systolic blood pressure, high sensitivity C-reactive protein, total cholesterol, HDL-cholesterol, hemoglobin A1c (%), current smoker, and family history of premature cardiovascular disease [18].
- The Guidelines of the International Task Force for Prevention of Coronary Disease propose the PROCAM calculator. It estimates the 10-year risk of major ischemic coronary disease or stroke, between 20 and 75 years of age for both sexes [19].
- In the United Kingdom, the National Institute for Health and Care Excellence (NICE) recommends the utilization of the QRISK2 for cardiovascular risk estimation [20].
- The Joint British Societies' consensus proposes the JBS3 risk calculator, based on the QRISK Lifetime. It adjusts a number of variables providing the probability of being alive and without cardiovascular disease at each age and the cumulative risk of cardiovascular disease [21].
- The Scottish Intercollegiate Guidelines Network recommend using the ASSIGN-SCORE, for estimation of the risk of cardiovascular events in individuals of 30–74 years of age [22].
- In Italy, the CUORE Project provides another risk estimator for the first major event in the next 10 years, based on gender, age, systolic blood pressure, total serum cholesterol, smoking status, and diabetes, applicable in primary prevention from 35 to 69 years of age [23].

All the models are valid, although they present several differences about what they are actually estimating and the way the result can be integrated in clinical practice. Two problems are often pointed to these calculators: the underestimation of the risk in younger individuals and the difficulty in the management of residual risk. Ageing is the main factor affecting the risk of cardiovascular disease. So, it is natural that even in the presence of other factors, the younger present low risk, especially if we make the calculations for the next 10 years. Estimating the cardiovascular risk age [24] is a way to overcome this difficulty. The concept is simple to explain and easy to visualize in a chart view. It may be useful in the younger particularly if the relative risk is high and even when the absolute risk is low. The long-term risk prediction algorithms also try to obviate this difficulty, but they are not fully established in clinical practice [25]. The sensitivity of Framingham Risk Score for coronary disease in the upper quintille is 45.9 in males and 57.5 in females and for stroke is 71.6 in males and 61.6 in females. The specificity is, respectively, 83.2 and 81.9 for coronary disease and 81.3 and 80.8 for stroke [15]. In prevention/screening, we need high sensitivity tests for detection of true negatives, and in diagnosis, we need high specificity tests to find the true positives. With these parameters, we ask what is the real advantage of these estimators in clinical practice.

Trying to solve some of these problems, the European Society of Cardiology developed the Systematic COronary Risk Evaluation (SCORE) based on a large number of European participants. The outcome variable is death by cardiovascular disease, chosen because it is a strong and reproducible variable. It allows separation of the mortality by ischemic heart disease and by stroke. Countries are categorized into low or high risk according to the mortality from the 45–74 age group by the cutoff of 225/100,000 in males and 175/100,000 in females, based on the 2012 data-CVD mortality rates of the WHO report [24]. The model provides for the possibility of calibration for each country according to the local specific mortality rate. Data were retrieved from 12 European cohort studies, including more than 250,000 patients and 3 million persons/year under observation, registering a total of about 7,000 deaths by cardiovascular disease [12]. However, current technology allows the survival of a significant proportion of patients with cardiovascular disease, limiting the interpretation of the final result of this calculation, once it only considers the deaths.

Although the model may present some variants, such as the utilization of cholesterol/HDL ratio, the variables included in the algorithm are age, sex, and 3 major risk factors (systolic blood pressure, total cholesterol, and current smoker) [12]. It also comprises several other modifiers with weight in the cardiovascular risk, allowing to lessen the error of the calculation: sedentary lifestyle, central obesity, poor socioeconomic conditions, low HDL-cholesterol, high triglycerides, fibrinogen, Apo-B, and increased lipoprotein (a), evidence of preclinical asymptomatic atherosclerosis (such as carotid plaques), presence of chronic kidney disease (Glomerular Filtration Rate—GFR < 60 mL/min/1.73 m^2^), and family history of premature cardiovascular death. It also considers the professionals' experience, including local conditioning, allowing correction of overestimation in places with decreasing cardiovascular mortality and underestimation in places of increasing incidence [26].

One interesting aspect of SCORE is its transposition into categories of risk rather than dealing with the absolute value of the calculation (Table 2). This categorization allows to simplify and personalize the characterization of each patient and to adjust the best intervention to each case [24].

Putting the continuous variable “risk” in a multinomial categorization, along with a broader approach by incorporating other significant elements, we abandon the vision of the estimator as a way to predict the future of the patient. Instead of trying to guess what will happen to our patient, this classification allows using current information as a tool for rational decision in the treatment of the several risk factors, including those that are not directly in the mathematical algorithm but are crucial to the decision reasoning.

This kind of approach simplifies the utilization of the decision flowchart in the complexity of the primary health care, where the provision of comprehensive and continuing care to every individual irrespective of age, sex, and illness [27], and implies to deal with several health problems at the same appointment and to find simultaneous responses to different demands [28]. It also makes the interpretation of the rating values easier. Regardless of the currently present factors, the risk will always be low in younger and high in elders, making it hard to establish the cutoff points above which a particular intervention or intensification would be mandatory, or, in the other hand, below which it would be unnecessary or potentially harmful, even if we were talking about a health counselling or a lifestyle intervention [24], which, of course, hardly makes any sense. Another difficulty is the utilization in youth people, where the absolute risk is usually low, giving a safety perspective not always completely true, even with the stratagem of using the concept of cardiovascular risk age.

Regardless of the discussion about the best formula to be used, the European Society of Cardiology recommends the evaluation of cardiovascular risk in all persons with a family history of premature cardiovascular disease, those with major risk factors, and those with significant comorbidities, with a maximum periodicity of 5 years. In the remaining population, asymptomatic and without known risk factors, risk assessment from the age of 40 in men and 50 years in women could be offered, although the evidence is less robust [24].

## 3. The Portuguese Situation

**Figure 3:**
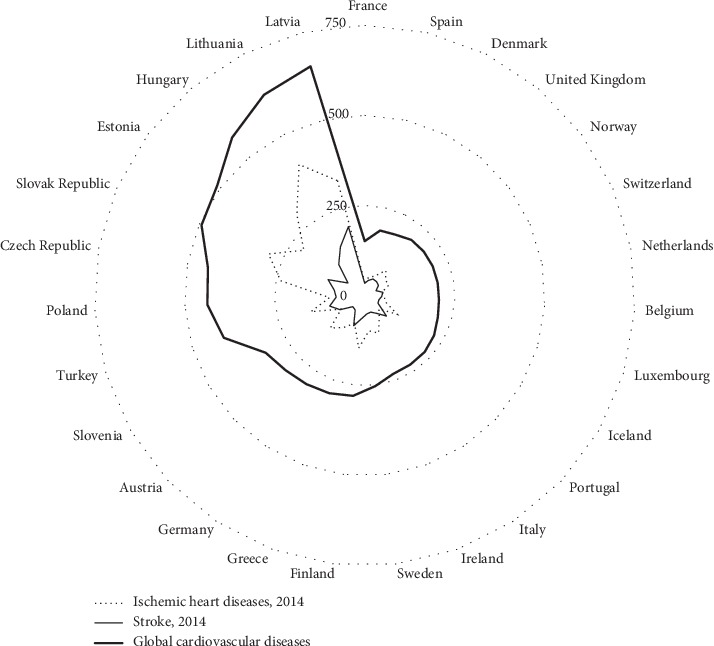
Standardized death rates by 100,000 people in 2014 in European countries (based on official data from OECD, 2018).

Mortality by cardiovascular diseases presents a heterogeneous distribution in Europe countries.  Figure 3 presents the standardized rates of deaths per 100,000 people, in 2014, according to OECD health data. With the exception of the Scandinavian countries, we may notice a gradient from north to south and from east to west.

Although Portugal is the most southwestern country in Europe, the relative position in cardiovascular death is medial, mainly because of stroke incidence. Portugal is one of the four OECD countries where mortality by stroke exceeds the mortality by ischemic heart disease. The importance of arterial hypertension over other risk factors, mostly related to salt consumption and to genetic factors, is a valid justification for it [29]. SCORE is adjusted for a scenario of prevailing ischemic heart disease, where Portugal is a low-risk country, based on age-adjusted cardiovascular mortality rates [24], but it does not allow the adjustment for stroke mortality, modifying the beta values of different risk factors. This restraint is similar in the other estimators, thus limiting its utilization.

Nevertheless, in a general point of view, the Bayes theorem tells us that a test is all the more decisive when the uncertainty is high, thus heightening the relevance of cardiovascular risk assessment in countries where cardiovascular diseases burden is medium.

The systematic evaluation of individual cardiovascular risk is proposed in the Portuguese national program for the cardio and cerebrovascular diseases, aiming to help to control the modifiable risk factors, especially hypertension and dyslipidemias. To consider an adequate follow-up, hypertensive patients must be assessed for global risk estimation at least each 3 years, using cardio-SCORE, which will be determinant for therapeutic decision making. The process is now at running and we cannot still have a real evaluation, despite the observed decrease of both mortality and hospital discharges by cardiovascular diseases in last years, mostly after 2007, when the current reorganization of primary health care was implemented, inserting a pay-per-performance approach in the remunerative regimen of health-care providers. The introduction of quality indicators to evaluate the system, although subject to much criticism, [30] may indeed improve the way we look to our patients at risk and manage them.

## 4. Conclusion

The continuity of care is a core characteristic of medical practice in primary health care, first in healthy and, from a certain point, also in the diseases that almost inevitably will appear during the lifetime. In the meantime, the risk factors emerge as the conditioners of the probability of developing clinical disease, depending on how they will express and how we can afford to manage them.

There are several algorithms to objectify the risk of cardiovascular diseases, given the diversity of factors at stake. None of them is sufficiently reliable to get a universal recommendation, especially if we continue to look at them from a divination point of view, as many times we see. Risk assessment allows us to weight the different factors, helping to categorize our patients from a cardiovascular perspective, aiming to prioritize the better decision towards a reduction of cardiovascular disease burden, both in the individual as well as in the whole population.

This risk modelling is crucial for preventive management, aiming to avoid the cardiovascular event, but with the concern of not causing harm and respecting the autonomy of the patient.

## Figures and Tables

**Table 1:** Risk factors for cardiovascular disease (adapted from Mendis et al. [6]).

Nonmodifiable
(i) Sex
(ii) Familial history
(iii) Genetic disposition
(iv) Race
(v) Age

Other modifiable risk factors
(i) Poverty
(ii) Psychological factors
(iii) Psychosocial stress
(iv) Alcohol abuse
(v) Some medications
(vi) Lipoprotein (a)
(vii) Left ventricular hypertrophy
Modifiable
(i) Arterial hypertension
(ii) Lipid disorders (LDL-cholesterol)
(iii) Tobacco
(iv) Overweight and Obesity
(v) Unhealthy diet
(vi) Sedentary
(vii) Diabetes mellitus
New risk factors
(i) Excess homocysteine
(ii) Inflammation
(iii) Disorders of blood coagulation

**Table 2:** Categories of cardiovascular risk, according to the European Society of Cardiology [24].

Very high risk	Documented cardiovascular disease
	Diabetes mellitus (type 1 or type 2) with one more risk factor or target organ damage
	Severe chronic kidney disease (GFR < 30 mL/min/1.73 m^2^); SCORE ≥ 10%

High risk	Markedly elevated risk factor (very high cholesterol or very high blood pressure)
	Diabetes mellitus (type 1 or type 2) without other risk factors or target organ damage
	Chronic kidney disease moderate (GFR = 30—59 mL/min/1.73 m^2^)
	SCORE ≥ 5% *e* < 10%
Moderate risk	SCORE ≥ 1% and <5%
Low risk	SCORE < 1%

GFR-Glomerular Filtration Rate.
